# Lobectomy versus segmentectomy for stage IA3 (T1cN0M0) non-small cell lung cancer: a meta-analysis and systematic review

**DOI:** 10.3389/fonc.2023.1270030

**Published:** 2023-10-02

**Authors:** Wanfei Zhang, Shaogeng Chen, Xianzuan Lin, Hongbo Chen, Rongqi He

**Affiliations:** Department of Thoracic Surgery, Quanzhou First Hospital Affiliated to Fujian Medical University, Quanzhou, Fujian, China

**Keywords:** lobectomy, segmentectomy, survival, non-small cell lung cancer, meta-analysis

## Abstract

**Background:**

Segmentectomy has been proven to have better survival and perioperative efficacy than lobectomy for non-small cell lung cancer (NSCLC) up to 2 cm. Whether this result is applicable to stage T1cN0M0 NSCLC (2.1 to 3 cm) remains controversial.

**Methods:**

We conducted a comprehensive search across seven databases to identify relevant studies comparing lobectomy and segmentectomy procedures. Our primary focus was on survival indicators (overall survival [OS] and disease-free survival [DFS]), while for secondary outcomes, operative outcomes, hospitalization outcomes, recurrences, and complications were considered.

**Results:**

After screening, the final analysis included 10 studies (involving 22113 patients in the lobectomy group and 1627 patients in the segmentectomy group). The lobectomy procedure achieved better OS (hazard ratio [HR]: 1.19 [1.07~1.33]) and DFS (HR: 1.37 [1.10~1.71]), which were proven in all subgroups. The OS rate at 2-5 years and DFS rate at 4-5 years were higher in the lobectomy group. The advantages of OS and DFS in the lobectomy group increased over the survival time. More lymph node dissections, intraoperative blood loss and total complications were found in the lobectomy group. Similar hospital stays, 90-day mortality and conversion thoracotomy were found between the two groups.

**Conclusion:**

Lobectomy appeared to be the better choice for patients with stage T1cN0M0 NSCLC with better survival (OS and DFS). However, the complications needed to be taken seriously.

**Systematic review registration:**

https://www.crd.york.ac.uk/PROSPERO/, identification CRD42023445013.

## Introduction

The incidence and mortality of lung cancer has been increasing over the past decades (1, 2). For stage IA non-small cell lung cancer (NSCLC), surgery remains the standard treatment method (2). In traditional concepts, lobectomy is the standard surgical procedure for these patients. However, in recent years, with the introduction of minimally invasive concepts, how to protect lung function as much as possible under the same survival efficacy has received attention from thoracic surgeons around the world (3). For stage IA1-2 (T1a-bN0M0) NSCLC, segmentectomy has been proven to have better perioperative efficacy, lung function protection, and noninferior survival efficacy compared with lobectomy (4–6). However, whether this conclusion is valid in stage IA3 NSCLC remains controversial in clinical practice.

To clarify this debate, several studies have been conducted in the past decade. Ogawa et al.’s and Deng et al.’s studies suggested that lobectomy achieved better overall survival (OS) and disease-free survival (DFS) than segmentectomy (7, 8). However, Forster et al.’s and Wang et al.’s studies did not find a survival advantage in the lobectomy group, and there were more complications in the lobectomy group (9, 10). Kamigaichi et al.’s and Yamashita et al.’s studies did not find any differences in the efficacy of survival and safety between the two groups (11, 12).

To clarify this controversy, we compared the survival and perioperative outcomes between lobectomy and segmentectomy procedures.

## Materials and methods

### Search strategy

This study adhered to the PRISMA guidelines (meta-analysis) (**Table S1**) (13). The study was preregistered on PROSPERO (ID: CRD42023445013). Then, we systematically searched seven databases (including Web of Science, EMBASE, etc.) until May 25, 2023. MeSH terms such as “lobectomy,” “segmentectomy,” and “lung cancer” were employed. The retrieval strategies can be found in **Table S2**. Additionally, we conducted a thorough search of the references in the retrieved literature to identify relevant articles.

#### Inclusion and exclusion criteria

Studies had to meet the following inclusion criteria:

(1) Population: patients diagnosed with stage IA3 (T1cN0M0) NSCLC based on The Eighth Edition Lung Cancer Stage Classification (14).
(2) Intervention and comparison: lobectomy *vs.* segmentectomy.
(3) Outcomes: survival, intraoperative outcomes, hospitalization outcomes, recurrences, and complications.
(4) Study design: randomized controlled trial (RCT) or cohort study.

When the same patient populations were involved in 2 or more studies, RCT and propensity score matching study would be prioritized, and if not, study with the largest sample size should be prioritized for inclusion. Animal experiments, meta-analyses, letters, commentaries, and reviews were excluded.

### Data extraction

Two independent investigators extracted the following data: baseline characteristic data, survival data (OS and DFS), intraoperative outcomes (operative time, etc.), recurrences (total, locoregional, and distant), hospitalization outcomes (hospital stay, etc.), and complications.

#### Outcome assessments

At 1-5 years, overall survival rate (OSR) and disease-free survival rate (DFSR) were analyzed. Subgroup analyses of OS and DFS were also conducted based on factors such as publication year, nation, stage, and data sources.

### Quality assessment

To evaluate the quality of cohort studies, we employed the Newcastle-Ottawa Scale (NOS), which incorporates three elements: comparability, selection, and outcome. High-quality studies scored 8 or 9 points and medium-quality studies scored 6 or 7 points (15).

Therefore, the highest-quality study would score 9 points. In our analysis, high-quality studies were defined as those that scored 8 or 9 points, and medium-quality studies were those that scored 6 or 7 points.

To assess the evidence level of the results, the Grades of Recommendations Assessment, Development, and Evaluation (GRADE) was utilized, which incorporated five components: publication bias, inconsistency, indirectness, risk of bias, and imprecision. Four levels of evidence existed: high, moderate, low, and very low (16).

### Statistical analysis

Review Manager 5.3 and STATA 12.0 were utilized for data analysis. Survival data were evaluated using hazard ratios (HRs), with HR > 1 indicating support for the lobectomy group. Meanwhile, the mean difference (MD) was used to analyze continuous variables, and the risk ratio (RR) was used to analyze dichotomous variables. The corresponding 95% confidence interval (95%CI) was calculated, and statistical significance was set at P<0.05. Heterogeneity was evaluated using the *I* ^2^ statistic and χ2 test. When heterogeneity was acceptable (*I* ^2^ < 50%), a fixed-effects model was employed. Conversely, a random-effects model was employed. Funnel plots (17), Egger’s test (18), and Begg’s test (19) were conducted to assess publication bias. Sensitivity analyses were performed by omitting individual studies to determine whether results were dependent on a single study (20).

## Results

### Study characteristics

**Figure 1:**
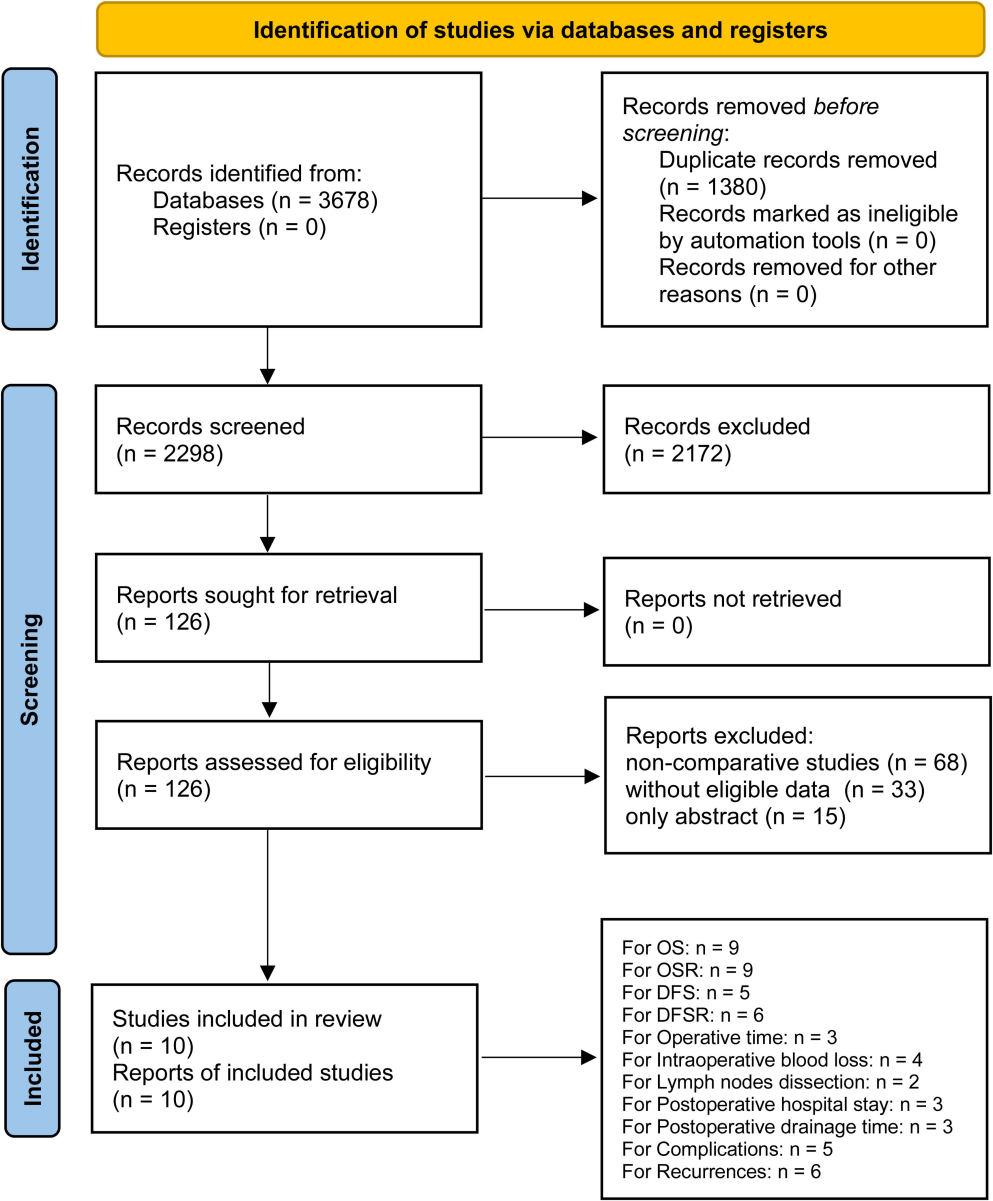
Flow chart of the study selection process.

After careful screening of 3678 studies, 10 studies met the inclusion criteria (7–12, 21–24) (**Figure 1**). The baseline characteristics are summarized in **Table 1**. Among them, five studies were performed in Asia (7, 11, 12, 21, 23), and five studies were performed in America and Europe (8–10, 22, 24). According to the NOS, 6 studies (9–12, 22, 23) were of high quality, and 4 studies (7, 8, 21, 24) were of medium quality (**Table S3**). The quality of evidence assessed using the GRADE was found to be low to very low for all outcomes (**Table S4**).

**Table 1:** Baseline characteristics of included literatures.

Study		Country	Period (year)	Groups	Patients	Sex (M/F)	Age (Mean, year)	Stage	Histology			Lesion location (lobes)					Follow up (months)
									SCC	AC	others	Right			Left		
												Upper	Middle	Lower	Upper	Lower	
2023	Forster (9)	Switzerland	2014-2021	Lobectomy	81	39/42	68	Pathologic T1cN0M0	19	60	2	33	0	16	21	11	32
				Segmentectomy	81	40/41	71		16	65	1	24	0	11	31	15	30
2022	Wang (10)	USA	2004-2015	Lobectomy	503	247/256	69.6	Pathologic T1cN0M0	124	187	192	254			249		45
				Segmentectomy	252	114/138	69.8		50	95	107	125			127		48
2022	Soh (21)	Japan	–	Lobectomy	1871	1104/767	–	Clinical T1cN0M0	–	1454	417	–	–	–	–	–	66.5
				Segmentectomy	129	78/51	–		–	121	8	–	–	–	–	–
2022	Peng (22)	USA	2010-2016	Lobectomy	18990	9153/9837	–	Pathologic T1cN0M0	6134	12192	665	11014			7976		–
				Segmentectomy	945	440/505	–		332	581	32	415			530		–
2022	Kadeetham (23)	Thailand	2016-2020	Lobectomy	60	24/36	65.3	Pathologic T1cN0M0	–	–	–	–	–	–	–	–	–
				Segmentectomy	17	7/10	68.5		–	–	–	–	–	–	–	–	–
2021	Chan (24)	USA	2013-2016	Lobectomy	279	132/147	68.8	Clinical T1cN0M0	83	178	18	169			109		60
				Segmentectomy	90	44/46	71.5		25	60	5	47			43		56.4
2020	Kamigaichi (11)	Japan	2007-2017	Lobectomy	37	19/18	71	Clinical T1cN0M0	8	22	7	5	0	12	12	8	42.2
				Segmentectomy	37	19/18	69		4	28	5	6	0	11	16	4
2015	Ogawa (7)	Japan	1994-2005	Lobectomy	147	85/62	63.9	Clinical T1cN0M0	24	123	0	63	0	31	25	28	93.4
				Segmentectomy	31	19/12	65.2		9	22	0	3	0	0	20	8
2014	Deng (8)	USA	1997-2012	Lobectomy	93	48/45	71.4	Pathologic T1cN0M0	21	53	19	58			35		–
				Segmentectomy	31	17/14	71.5		5	18	8	15			16		–
2012	Yamashita (12)	Japan	2003-2011	Lobectomy	52	26/26	68	Pathologic T1cN0M0	8	21	23	–	–	–	–	–	–
				Segmentectomy	14	6/8	69		2	4	8	–	–	–	–	–	–

AC, adenocarcinoma; M/F, male/female; TNM, Tumor Node Metastasis; SCC, squamous cell carcinoma.

a Pathological TNM stage was according to 8th edition of TNM classification.

#### Survival

**Figure 2:**
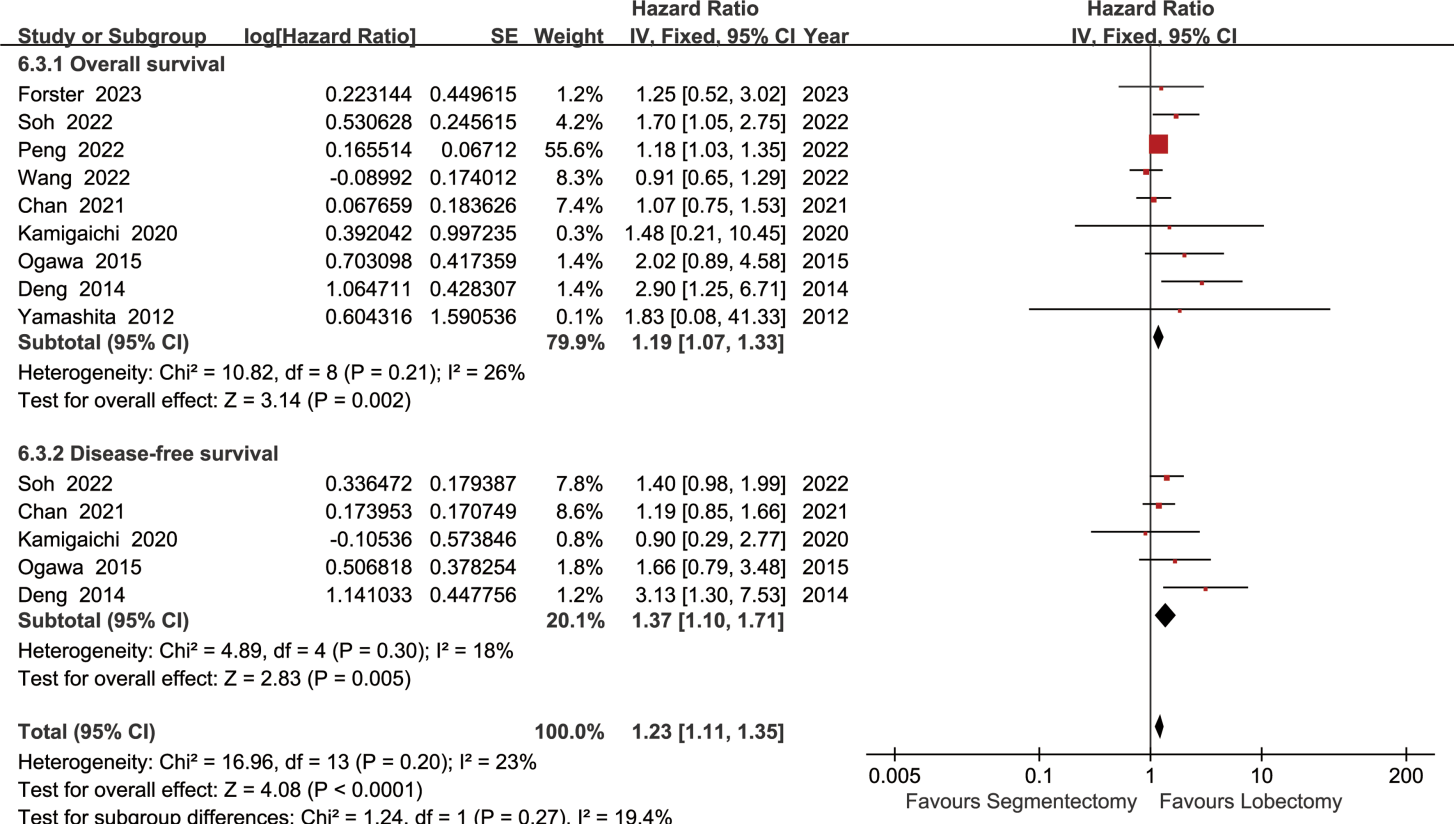
Forest plots of overall survival and disease-free survival associated with lobectomy versus segmentectomy.

**Figure 3:**
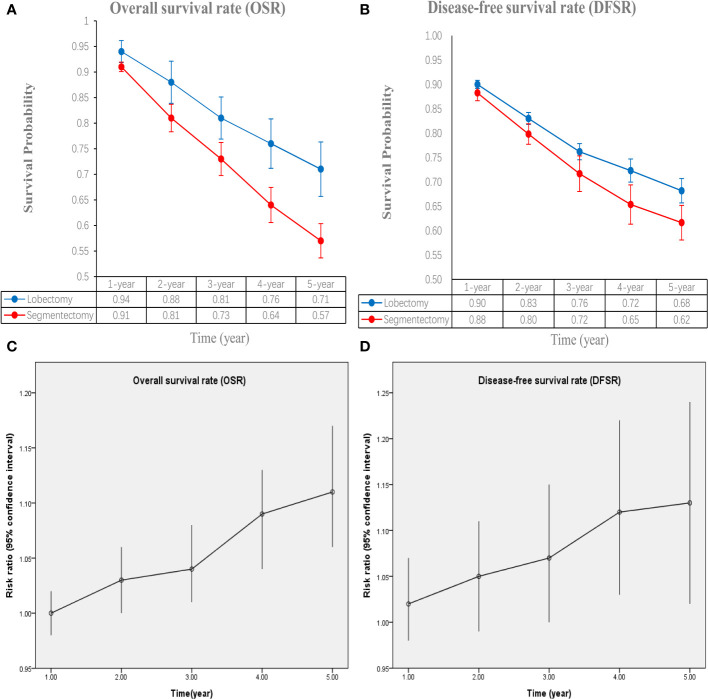
Comparisons of overall survival rate (1-5 years, **A**: trend of overall survival rate; **C**: trend of risk ratios) and disease-free survival rate (1-5 years, **B**: trend of disease-free survival rate; **D**: trend of risk ratios) associated with lobectomy versus segmentectomy according to survival time.

The lobectomy group showed better OS than the segmentectomy group (HR: 1.19 [1.07~1.33], **Figure 2**). Subgroup analyses indicated that the lobectomy group had a higher OSR at 2 years (RR: 1.03 [1.00~1.06]), 3 years (RR: 1.04 [1.01~1.08]), 4 years (RR: 1.09 [1.04~1.13]) and 5 years (RR: 1.11 [1.06~1.17]) (**Figures S1**, **3A**). The OSR in the lobectomy group showed an increasing trend over time (**Figure 3C**).

The lobectomy group showed better DFS than the segmentectomy group (HR: 1.37 [1.10~1.71], **Figure 2**). Subgroup analyses indicated that the lobectomy group had a higher DFSR at 4 years (RR: 1.12 [1.03~1.22]) and 5 years (RR: 1.13 [1.02~1.24]) (**Figures S2**, **3B**). The DFSR in the lobectomy group showed an increasing trend over time (**Figure 3D**).

The two groups had similar total recurrences (RR: 1.27 [0.62~2.62]), locoregional recurrences (RR: 1.11 [0.46~2.68]) and distant recurrences (RR: 1.44 [0.60~3.48]) (**Figure S3**).

### Subgroup analysis of survival

Subgroup analyses were performed according to published year, nation, stage, and data sources. The survival advantages of the lobectomy group were achieved in all subgroups (**Table 2**).

**Table 2:** Subgroup analysis of overall survival and disease-free survival associated with lobectomy versus segmentectomy.

Subgroups	No. of studies	Overall Survival		No. of studies	Disease-Free Survival	
		HR (95% CI)	*P*		HR (95% CI)	*P*
**Total**	9	1.19 (1.07-1.33)	0.002	5	1.37 (1.10-1.71)	0.005
Published year						
Earlier than 2020	3	2.39 (1.34-4.24)	0.003	2	2.16 (1.23-3.81)	0.008
2020-	6	1.16 (1.04-1.30)	0.009	3	1.27 (1.00-1.60)	0.05
Nation						
Asia	4	1.76 (1.18-2.64)	0.006	3	1.39 (1.03-1.89)	0.03
Europe and America	5	1.16 (1.03-1.30)	0.001	2	1.35 (0.98-1.84)	0.06
Stage						
Pathologic	5	1.17 (1.03-1.32)	0.01	1	3.13 (1.30-7.53)	0.01
Clinical	4	1.33 (1.02-1.75)	0.04	4	1.30 (1.04-1.63)	0.02
Data sources						
Hospital	6	1.33 (1.00-1.77)	0.05	4	1.35 (1.02-1.79)	0.03
Database	3	1.17 (1.04-1.32)	0.01	1	1.40 (0.98-1.99)	0.06

CI, confidence interval; HR, hazard ratio; No., number.

When the HR > 1, the results supported the lobectomy group.

#### Intraoperative indicators

**Figure 4:**
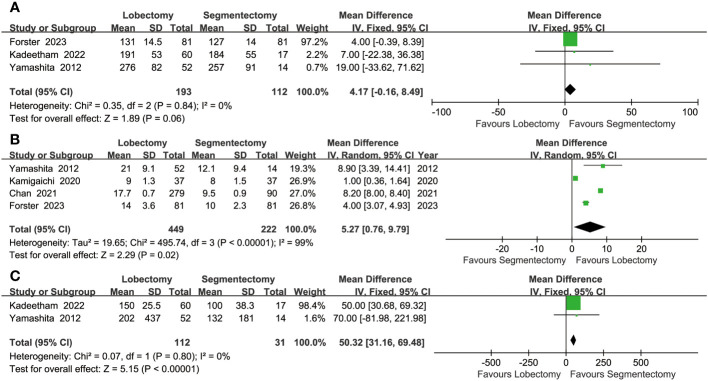
Forest plots of operative time **(A)** lymph nodes dissection **(B)** and intraoperative blood loss **(C)** associated with lobectomy versus segmentectomy.

More lymph node dissections (MD: 5.27 [0.76~9.79], *p* = 0.02, **Figure 4B**) and intraoperative blood loss (MD: 50.32 [31.16~69.48] ml, *p* < 0.00001, **Figure 4C**) were found in the lobectomy group. The segmentectomy group tended to have a more favorable operative time (MD: 4.17 [-0.16~8.49] minutes, *p* = 0.06, **Figure 4A**), without statistical significance.

#### Hospitalization indicators

**Figure 5:**
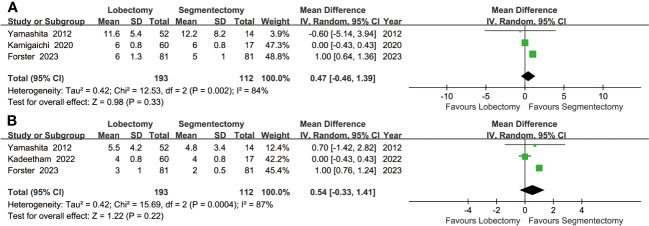
Forest plots of postoperative hospital stay **(A)** and postoperative drainage time **(B)** associated with lobectomy versus segmentectomy.

Similar drainage times (MD: 0.54 [-0.33~1.41] days**)** and hospital stays (MD: 0.47 [-0.46~1.39] days**)** were found between the two groups (**Figure 5**).

#### Complications

More total complications were found in the lobectomy group (RR: 1.28 [1.04~1.59], *p* = 0.02). Similar severe complications, 90-day mortality, conversion thoracotomy, pulmonary complications, cardiac complications, reoperation, readmission, atrial fibrillation, air leak >5 days, postoperative bleeding, acute renal failure, urinary retention, acute myocardial infarction, embolism, chylothorax, empyema and wound infection were found between the groups (**Figure S4**).

#### Sensitivity analysis

A sensitivity analysis was performed for comparisons with high heterogeneity (recurrences, lymph node dissection, postoperative hospital stays and drainage time). The RR/HR/MD and 95% CI did not change significantly after removal of any single study, which indicated that the results were stable (**Figure S5**).

#### Publication bias

**Figure 6:**
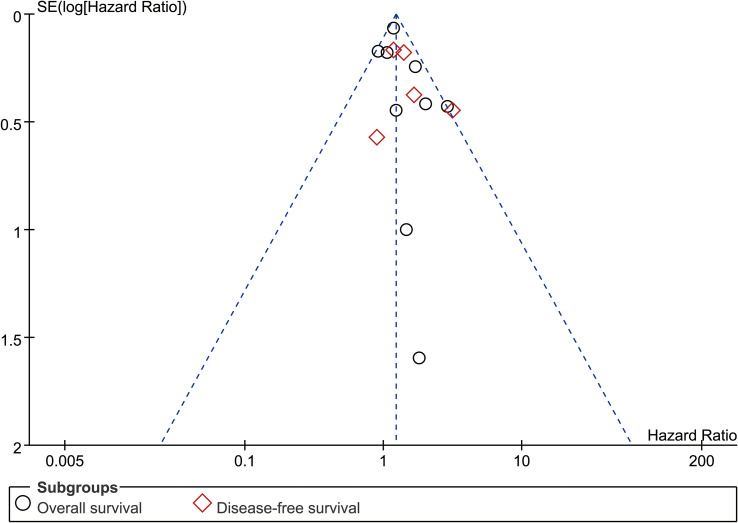
Funnel plot of overall survival and disease-free survival.

Funnel plots (OS and DFS) showed no significant publication bias, which was also confirmed by Egger’s and Begg’s tests (**Figures 6**, **S6**).

## Discussion

For a long time, lobectomy is the standard surgical procedure for stage I NSCLC (25). In recent years, some evidence has shown that segmentectomy has better outcomes than lobectomy in stage IA1-2 (T1a-bN0M0) NSCLC (4–6). However, whether this conclusion is valid in stage IA3 (T1cN0M0) NSCLC remains controversial in clinical practice. This meta-analysis compared the two surgical procedures in IA3 (T1cN0M0) NSCLC. The results suggested that the lobectomy group achieved better OS and DFS. The survival advantages in the lobectomy group increased over the survival time. More lymph nodes dissection, intraoperative blood loss and complications were found in lobectomy group.

Better survival is the main advantage with lobectomy, and this advantage will increase with the prolongation of survival. The OSR-5y is 71% in the lobectomy group and 57% in the segmentectomy group. Tendency for survival advantage were supported by six included studies (7–9, 11, 12, 21). Yu et al.’s study based on 9580 patients also suggested that lobectomy and complete lymph node dissection should be the recommended standard of care for patients with stage IA3 NSCLC (26). Three reasons may explain this result: (1) Farther tumor margins reduce the possibility of local tumor recurrence in the lobectomy group (8); (2) More lymph node dissection numbers reduce the lymph node recurrence in the lobectomy group (12, 24); (3) Some N1 lymph nodes were not removed in the segmentectomy group, which may affect the staging judgment. Higher actual pathologic staging may affect the prognosis of patients in the segmentectomy group (7). Meanwhile, the survival advantage of lobectomy was proved in all subgroups according to the published year, nation, stage, and data sources. Therefore, we believe that lobectomy should be performed for stage IA3 NSCLC, which not only meets the requirements of the guidelines but also meets the survival needs of patients

Surgical safety is another important indicator for evaluating surgery. Lower intraoperative and postoperative complications often indicate better quality of life and lower costs (27). In this study, more operative time and intraoperative blood loss were found during lobectomy procedure, which is consistent with the actual situation in our surgeries. From the specific data, an average bleeding increment of 50.22 ml and a 4.17 minutes surgical time increment per surgery are acceptable by most patients and surgeons. Meanwhile, more total complications were found in the lobectomy group (RR: 1.28 [1.04~1.59]), which is also in line with our actual postoperative situation. Similar results were also reported in the Ichinose et al.’s study based on 59663 patients (28). However, no significant difference was found in the comparison of all single complications. Pulmonary complications and atrial fibrillation are the two most common complications after lung surgery, with incidence rates of approximately 20% in each group, respectively. Meanwhile, age, operation time and number of lymph node dissected during operation are independent risk factors affecting postoperative complications (29). Thus, although lobectomy may result in better survival outcomes, its more frequent complications need to be taken seriously.

The main consideration for choosing segmentectomy is to protect lung function as much as possible. For NSCLC up to 2 cm, Bao et al. (30) and Ji et al. (29) reported that segmentectomy shows less loss of lung function than lobectomy. Xu et al. (31) suggested that segmentectomy is helpful to minimize the loss of FVC, but not FEV1 or DLCO. However, for tumors of this size (2-3 cm), in order to ensure sufficient distance between the tumor margins, segmental resection of lung tissue is often larger than single lung segment, and even requires combined segmental resection. In these patients, it is controversial whether lung function could be protected, as compared to lobectomy (32). In the current study, due to database limitations, no analysis results related to lung function protection have been obtained, which is important in the future research.

There are still some limitations in the study. First, all retrieved databases are in English, which might result in some non-English published papers that meet the standards not being included. Second, the evidence level of all results is low or very low, which may reduce the credibility of the conclusion. Third, data of lung function was lacking in all the included studies, and it is important to compare the two surgery procedures. Fourth, there are two forms of staging: clinical staging and pathological staging, which might increase the heterogeneity. Fifth, the data sources are different because some of the data were from large databases and some were from single centers, which might increase the heterogeneity.

## Conclusion

In summary, lobectomy appeared to be the better choice for patients with stage IA3 NSCLC with better survival (OS and DFS). The survival advantages in the lobectomy group increased over the survival time. Meanwhile, more attention should be given to the control of postoperative complications. However, due to insufficient evidence of the results, large sample RCTs need to be conducted to confirm the conclusion.

## Data availability statement

The original contributions presented in the study are included in the article/**Supplementary Material**. Further inquiries can be directed to the corresponding author.

## Author contributions

WZ: Conceptualization, Data curation, Formal Analysis, Methodology, Resources, Software, Writing – original draft, Writing – review & editing. SC: Conceptualization, Data curation, Formal Analysis, Investigation, Methodology, Writing – original draft. XL: Conceptualization, Data curation, Formal Analysis, Investigation, Methodology, Software, Writing – original draft. HC: Conceptualization, Data curation, Formal Analysis, Investigation, Methodology, Software, Writing – original draft. RH: Conceptualization, Data curation, Formal Analysis, Funding acquisition, Investigation, Methodology, Project administration, Resources, Software, Supervision, Validation, Visualization, Writing – original draft, Writing – review & editing.

## Supplementary material

Supplementary Figure 1 Comparisons of overall survival rate (1-5 years) associated with lobectomy versus segmentectomy.

Supplementary Figure 2 Comparisons of disease-free survival rate (1-5 years) associated with lobectomy versus segmentectomy.

Supplementary Figure 3 Comparisons of recurrences associated with lobectomy versus segmentectomy.

Supplementary Figure 4 Forest plots of complications associated with lobectomy versus segmentectomy.

Supplementary Figure 5 Sensitivity analysis of recurrences **(A)**, lymph nodes dissection **(B)**, postoperative hospital stay **(C)** and postoperative drainage time **(D)**.

Supplementary Figure 6 Egger’s and Begg’s tests of overall survival **(A)** and disease-free survival **(B)**.
