# Supplementary figures and images for: Lobectomy versus segmentectomy for stage IA3 (T1cN0M0) non-small cell lung cancer: a meta-analysis and systematic review
Source: Front Oncol. 2023 Oct 2;13:1270030. doi: 10.3389/fonc.2023.1270030 (PMC10578965; doi:10.3389/fonc.2023.1270030)

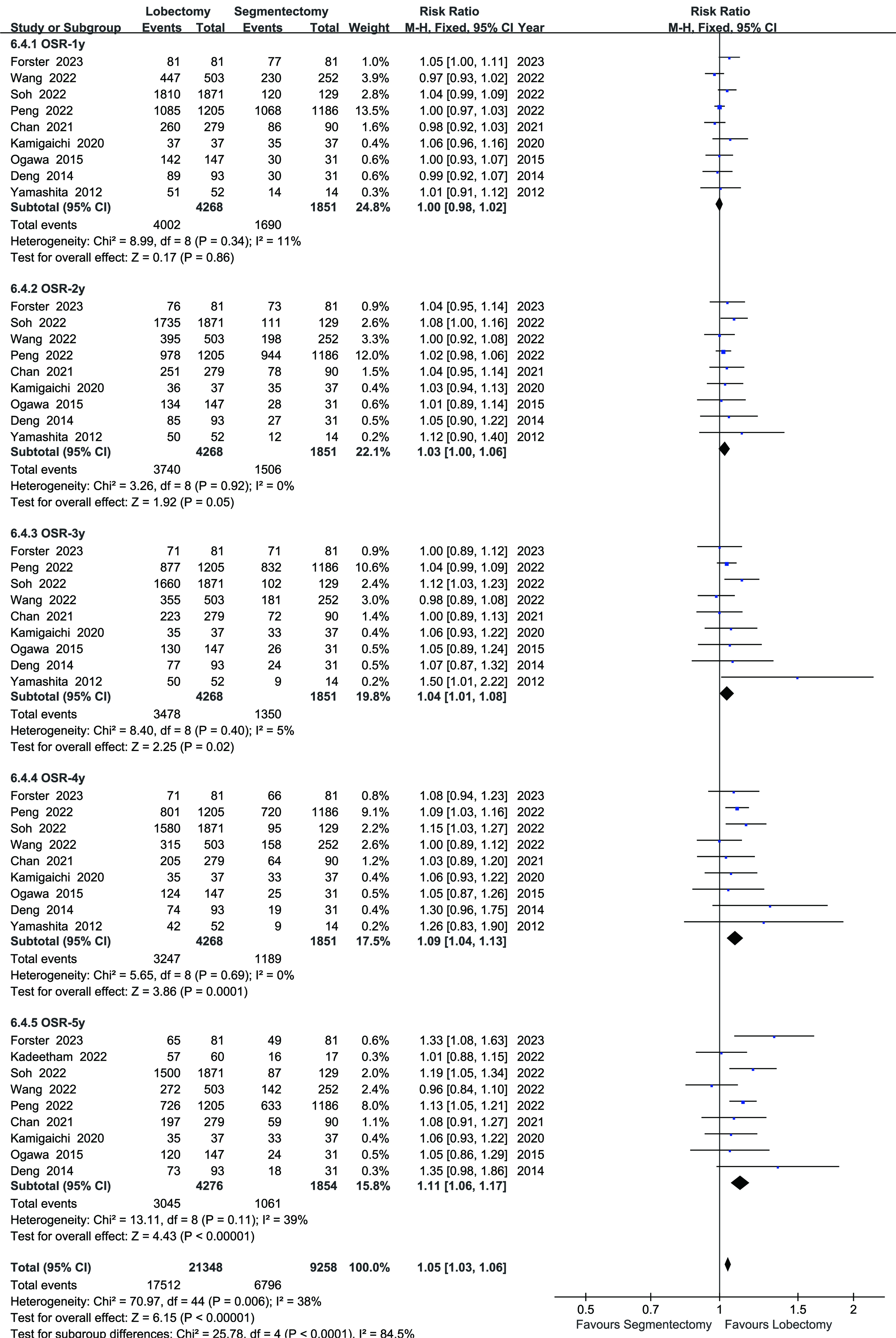

Supplement: Supplementary Figure 1 — Comparisons of overall survival rate (1-5 years) associated with lobectomy versus segmentectomy. [file Image_1.tif]

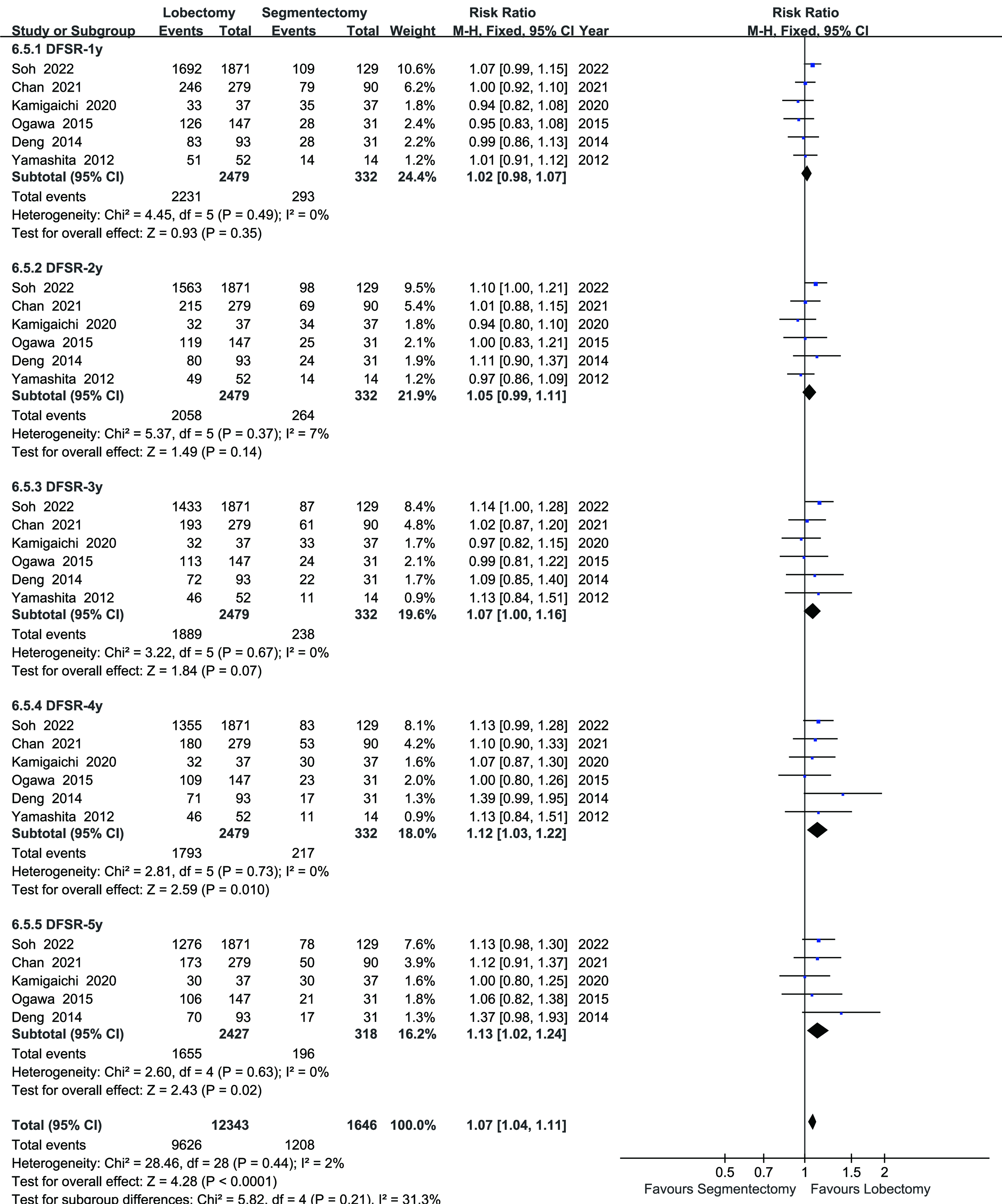

Supplement: Supplementary Figure 2 — Comparisons of disease-free survival rate (1-5 years) associated with lobectomy versus segmentectomy. [file Image_2.tif]

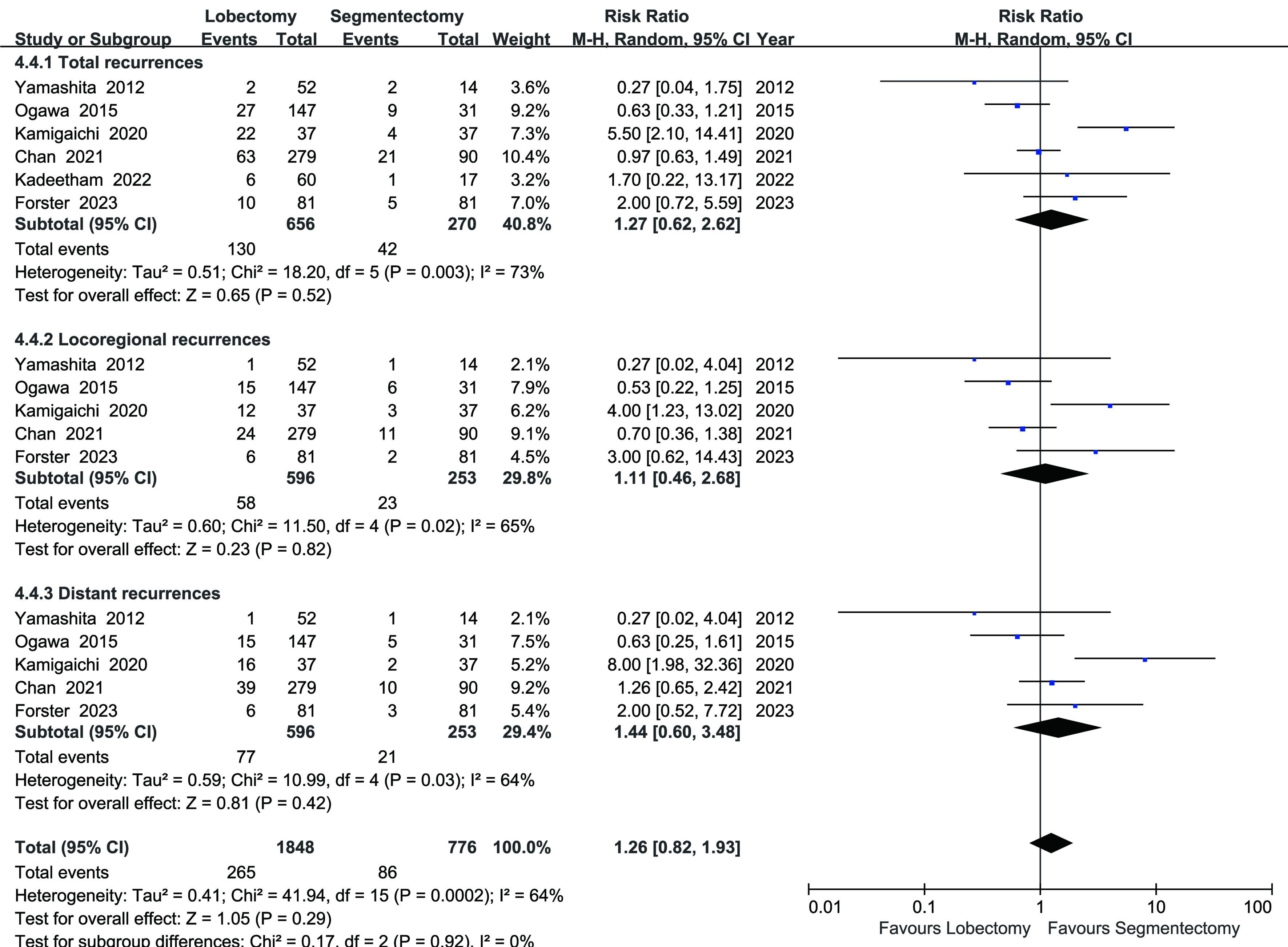

Supplement: Supplementary Figure 3 — Comparisons of recurrences associated with lobectomy versus segmentectomy. [file Image_3.tif]

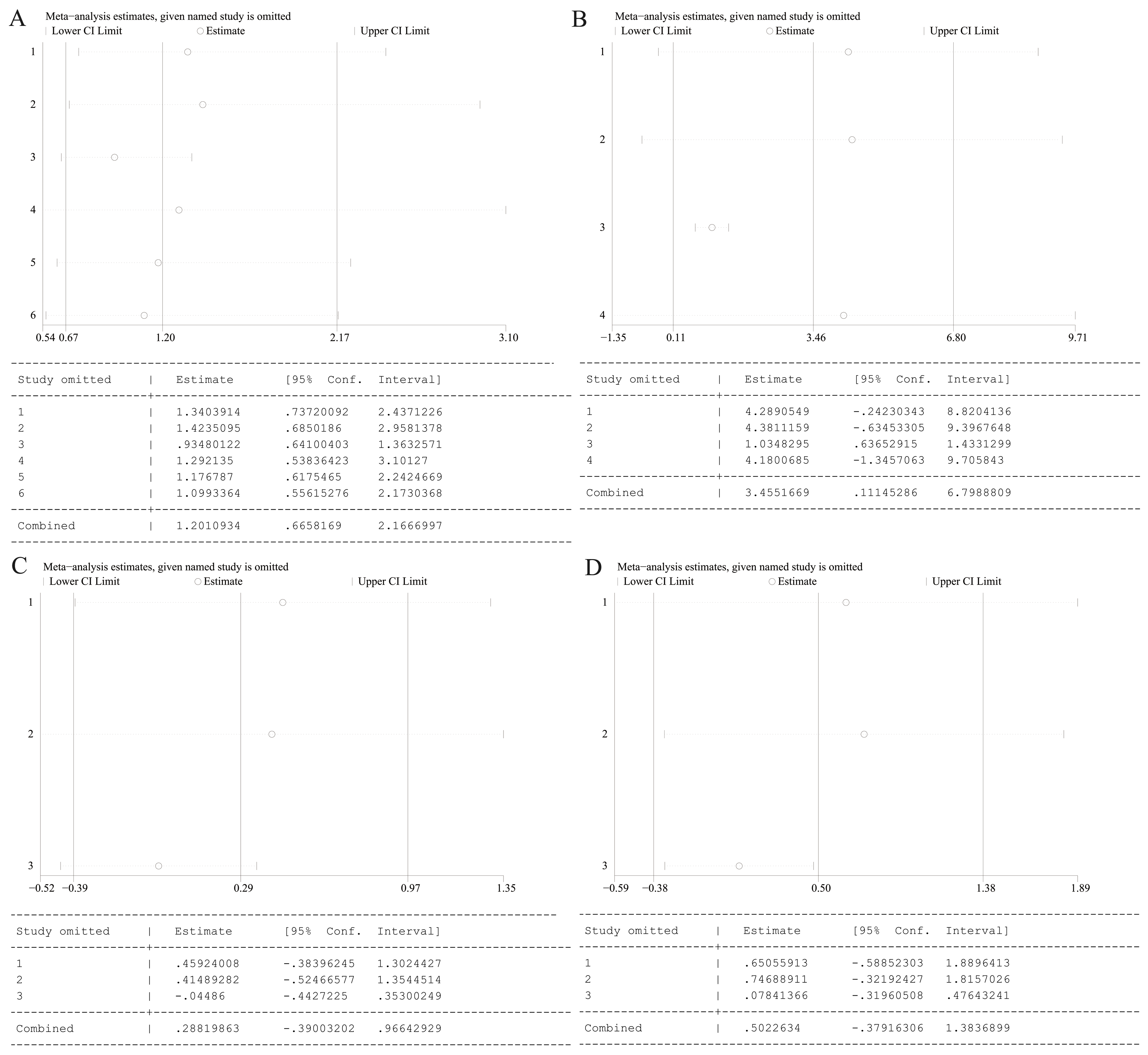

Supplement: Supplementary Figure 5 — Sensitivity analysis of recurrences (A), lymph nodes dissection (B), postoperative hospital stay (C) and postoperative drainage time (D). [file Image_5.tif]

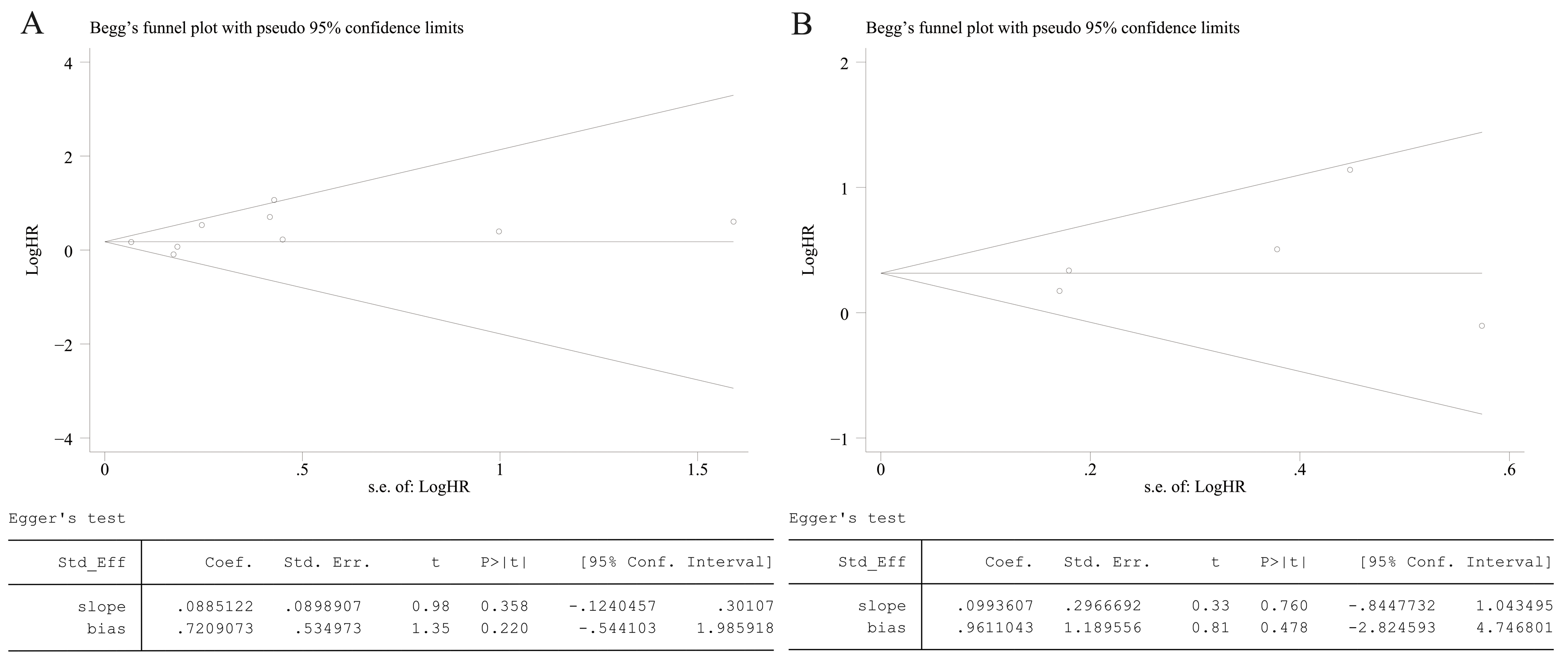

Supplement: Supplementary Figure 6 — Egger’s and Begg’s tests of overall survival (A) and disease-free survival (B). [file Image_6.tif]
