# Supplementary material for: Lobectomy versus segmentectomy for stage IA3 (T1cN0M0) non-small cell lung cancer: a meta-analysis and systematic review
Source: Front Oncol. 2023 Oct 2;13:1270030. doi: 10.3389/fonc.2023.1270030 (PMC10578965; doi:10.3389/fonc.2023.1270030)
Supplement: Supplementary file 8 [file Table_2.docx]

**Table S2** Search strategy

| **PubMed**  The database was searched on May 25, 2023, n=282.  Search Strategy:  (Segmentectomy[Title/Abstract]) AND (Lobectomy[Title/Abstract]) AND (Lung neoplasms[Title/Abstract] OR Pulmonary Neoplasms[Title/Abstract] OR Neoplasms, Lung[Title/Abstract] OR Lung Neoplasm[Title/Abstract] OR Neoplasm, Lung[Title/Abstract] OR Neoplasms, Pulmonary[Title/Abstract] OR Neoplasm, Pulmonary[Title/Abstract] OR Pulmonary Neoplasm[Title/Abstract] OR Lung Cancer[Title/Abstract] OR Cancer, Lung[Title/Abstract] OR Cancers, Lung[Title/Abstract] OR Lung Cancers[Title/Abstract] OR Pulmonary Cancer[Title/Abstract] OR Cancer, Pulmonary[Title/Abstract] OR Cancers, Pulmonary[Title/Abstract] OR Pulmonary Cancers[Title/Abstract] OR Cancer of the Lung[Title/Abstract] OR Cancer of Lung[Title/Abstract]) |
| --- |
| **Web of Science**  The database was searched on May 25, 2023, n=564.  Search Strategy:  1 TOPIC: (“Segmentectomy”)  2 TOPIC: (“Lobectomy”)  3 TOPIC: (“Lung neoplasms” OR “Pulmonary Neoplasms” OR “Neoplasms, Lung” OR “Lung Neoplasm” OR “Neoplasm, Lung” OR “Neoplasms, Pulmonary” OR “Neoplasm, Pulmonary” OR “Pulmonary Neoplasm” OR “Lung Cancer” OR “Cancer, Lung” OR “Cancers, Lung” OR “Lung Cancers” OR “Pulmonary Cancer” OR “Cancer, Pulmonary” OR “Cancers, Pulmonary” OR “Pulmonary Cancers” OR “Cancer of the Lung” OR “Cancer of Lung”)  4 #1 AND #2 AND #3 |
| **EMBASE**  The database was searched on May 25, 2023, n=745.  Search Strategy:  ('Segmentectomy':ti,ab,kw) AND ('Lobectomy':ti,ab,kw) AND ('Lung neoplasms':ti,ab,kw OR 'Pulmonary Neoplasms':ti,ab,kw OR 'Neoplasms, Lung':ti,ab,kw OR 'Lung Neoplasm':ti,ab,kw OR 'Neoplasm, Lung':ti,ab,kw OR 'Neoplasms, Pulmonary':ti,ab,kw OR 'Neoplasm, Pulmonary':ti,ab,kw OR 'Pulmonary Neoplasm':ti,ab,kw OR 'Lung Cancer':ti,ab,kw OR 'Cancer, Lung':ti,ab,kw OR 'Cancers, Lung':ti,ab,kw OR 'Lung Cancers':ti,ab,kw OR 'Pulmonary Cancer':ti,ab,kw OR 'Cancer, Pulmonary':ti,ab,kw OR 'Cancers, Pulmonary':ti,ab,kw OR 'Pulmonary Cancers':ti,ab,kw OR 'Cancer of the Lung':ti,ab,kw OR 'Cancer of Lung':ti,ab,kw) |
| **Cochrane Library**  The database was searched on May 25, 2023, n=167.  Search Strategy:  (“Segmentectomy”): ti,ab,kw AND (“Lobectomy”): ti,ab,kw AND (“Lung neoplasms” OR “Pulmonary Neoplasms” OR “Neoplasms, Lung” OR “Lung Neoplasm” OR “Neoplasm, Lung” OR “Neoplasms, Pulmonary” OR “Neoplasm, Pulmonary” OR “Pulmonary Neoplasm” OR “Lung Cancer” OR “Cancer, Lung” OR “Cancers, Lung” OR “Lung Cancers” OR “Pulmonary Cancer” OR “Cancer, Pulmonary” OR “Cancers, Pulmonary” OR “Pulmonary Cancers” OR “Cancer of the Lung” OR “Cancer of Lung”): ti,ab,kw - (Word variations have been searched) |
| **Ovid MEDLINE**  The database was searched on May 25, 2023, n=958.  Search Strategy:  1 Segmentectomy.ab.  2 Lobectomy.ab.  3 Pulmonary Neoplasms.ab.  4 Neoplasms, Lung.ab.  5 Lung Neoplasm.ab.  6 Neoplasm, Lung.ab.  7 Neoplasms, Pulmonary.ab.  8 Neoplasm, Pulmonary.ab.  9 Pulmonary Neoplasm.ab.  10 Lung Cancer.ab.  11 Cancer, Lung.ab.  12 Cancers, Lung.ab.  13 Lung Cancers.ab.  14 Pulmonary Cancer.ab.  15 Cancer, Pulmonary.ab.  16 Cancers, Pulmonary.ab.  17 Pulmonary Cancers.ab.  18 Cancer of the Lung.ab.  19 Cancer of Lung.ab.  20 or/3-19 [Lung cancer]  21 1 and 2 and 20 |
| **ScienceDirect**  The database was searched on May 25, 2023, n=105.  Search Strategy:  Title, abstract, keywords: ((“Segmentectomy”) and (“Lobectomy”) and (“Lung neoplasms” OR “Pulmonary Neoplasms” OR “Neoplasms, Lung” OR “Lung Neoplasm” OR “Neoplasm, Lung” OR “Neoplasms, Pulmonary” OR “Neoplasm, Pulmonary” OR “Pulmonary Neoplasm” OR “Lung Cancer” OR “Cancer, Lung” OR “Cancers, Lung” OR “Lung Cancers” OR “Pulmonary Cancer” OR “Cancer, Pulmonary” OR “Cancers, Pulmonary” OR “Pulmonary Cancers” OR “Cancer of the Lung” OR “Cancer of Lung”)) |
| **Scopus**  The database was searched on May 25, 2023, n=856.  Search Strategy:  TITLE-ABS-KEY ((“Segmentectomy”) and (“Lobectomy”) and (“Lung neoplasms” OR “Pulmonary Neoplasms” OR “Neoplasms, Lung” OR “Lung Neoplasm” OR “Neoplasm, Lung” OR “Neoplasms, Pulmonary” OR “Neoplasm, Pulmonary” OR “Pulmonary Neoplasm” OR “Lung Cancer” OR “Cancer, Lung” OR “Cancers, Lung” OR “Lung Cancers” OR “Pulmonary Cancer” OR “Cancer, Pulmonary” OR “Cancers, Pulmonary” OR “Pulmonary Cancers” OR “Cancer of the Lung” OR “Cancer of Lung”)) |

**Note:** The combined text and medical subject heading (MeSH) terms used were: “Segmentectomy”, “Lobectomy” and “Lung cancer”.
