# Supplementary material for: Lobectomy versus segmentectomy for stage IA3 (T1cN0M0) non-small cell lung cancer: a meta-analysis and systematic review
Source: Front Oncol. 2023 Oct 2;13:1270030. doi: 10.3389/fonc.2023.1270030 (PMC10578965; doi:10.3389/fonc.2023.1270030)
Supplement: Supplementary file 9 [file Table_3.doc]

**Table S3 Methodological quality assessments of the included studies.**

| **Study** | | Selection | | | | Comparability^d^ | Outcome | | | Total score |
| --- | --- | --- | --- | --- | --- | --- | --- | --- | --- | --- |
|  |  | Exposed cohort^a^ | Nonexposed cohort^b^ | Ascertainment of exposure | Outcome of interest^c^ |  | Assessment of outcome | Length of follow-up^e^ | Adequacy of follow-up |  |
| 2023 | Forster [9] | * |  | * | * | ** | * | * | * | 8 |
| 2022 | Wang [10] | * | * | * | * | ** |  | * | * | 8 |
| 2022 | Soh [21] | * |  | * | * | ** | * |  | * | 7 |
| 2022 | Peng [22] | * | * | * | * | ** |  | * | * | 8 |
| 2022 | Kadeetham [23] | * |  | * | * | ** | * | * | * | 8 |
| 2021 | Chan [24] |  | * | * | * | ** |  | * | * | 7 |
| 2020 | Kamigaichi [11] | * | * | * | * | ** | * | * | * | 9 |
| 2015 | Ogawa [7] | * |  | * | * | ** |  | * | * | 7 |
| 2014 | Deng [8] | * |  | * | * | ** | * |  | * | 7 |
| 2012 | Yamashita [12] | * | * | * | * | ** | * | * | * | 9 |

Note: ^a^ Representativeness of the exposed cohort;

^b^ Selection of the non-exposed cohort;

^c^ Demonstration that outcome of interest was not present at start of study;

^d^ Comparability of cohorts was based on the design or analysis;

^e^ Was follow-up long enough for outcomes to occur.
