# Supplementary material for: Lobectomy versus segmentectomy for stage IA3 (T1cN0M0) non-small cell lung cancer: a meta-analysis and systematic review
Source: Front Oncol. 2023 Oct 2;13:1270030. doi: 10.3389/fonc.2023.1270030 (PMC10578965; doi:10.3389/fonc.2023.1270030)
Supplement: Supplementary file 10 [file Table_4.doc]

**Table S4** GRADE quality assessment by therapeutic strategy and study design for the outcomes.

| **Primary outcomes** | **No. of Studies** | **No. of Participants** | | **Differences（95%CI）a** | **Quality Assessment** | | | | | **Quality** |
| --- | --- | --- | --- | --- | --- | --- | --- | --- | --- | --- |
|  |  | **Vein-First** | **Artery-First** |  | **Risk of Bias b** | **Inconsistency** | **Indirectness** | **Imprecision** | **Publication Bias c** |  |
| **Survival** |  |  |  |  |  |  |  |  |  |  |
| OS | 9 | 22053 | 1610 | 1.19 [1.07, 1.33] | Serious (-1) | No inconsistency | No indirectness | No imprecision | Unlikely | Very Low |
| OSR |  |  |  |  |  |  |  |  |  |  |
| 1-year | 9 | 4002/4268 | 1690/1851 | 1.00 [0.98, 1.02] | Low | No inconsistency | No indirectness | No imprecision | Unlikely | Low |
| 2-year | 9 | 3740/4268 | 1506/1851 | 1.03 [1.00, 1.06] | Low | No inconsistency | No indirectness | No imprecision | Unlikely | Low |
| 3-year | 9 | 3478/4268 | 1350/1851 | 1.04 [1.01, 1.08] | Low | No inconsistency | No indirectness | No imprecision | Unlikely | Low |
| 4-year | 9 | 3247/4268 | 1189/1851 | 1.09 [1.04, 1.13] | Low | No inconsistency | No indirectness | No imprecision | Unlikely | Low |
| 5-year | 9 | 3045/4276 | 1061/1854 | 1.11 [1.06, 1.17] | Low | No inconsistency | No indirectness | No imprecision | Unlikely | Low |
| DFS | 5 | 2427 | 318 | 1.37 [1.10, 1.71] | Serious (-1) | No inconsistency | No indirectness | No imprecision | Unlikely | Very Low |
| DFSR |  |  |  |  |  |  |  |  |  |  |
| 1-year | 6 | 2231/2479 | 293/332 | 1.02 [0.98, 1.07] | Low | No inconsistency | No indirectness | No imprecision | Unlikely | Low |
| 2-year | 6 | 2058/2479 | 264/332 | 1.05 [0.99, 1.11] | Low | No inconsistency | No indirectness | No imprecision | Unlikely | Low |
| 3-year | 6 | 1889/2479 | 238/332 | 1.07 [1.00, 1.16] | Low | No inconsistency | No indirectness | No imprecision | Unlikely | Low |
| 4-year | 6 | 1793/2479 | 217/332 | 1.12 [1.03, 1.22] | Low | No inconsistency | No indirectness | No imprecision | Unlikely | Low |
| 5-year | 5 | 1655/2427 | 196/318 | 1.13 [1.02, 1.24] | Low | No inconsistency | No indirectness | No imprecision | Unlikely | Low |
| **Operative outcomes** |  |  |  |  |  |  |  |  |  |  |
| Operative time | 3 | 193 | 112 | 4.17 [-0.16, 8.49] | Very Serious (-2) | Very Serious (-2) | No indirectness | No imprecision | Unlikely | Very Low |
| Intraoperative blood loss | 4 | 449 | 222 | 5.27 [0.76, 9.79] | Unclear | No inconsistency | No indirectness | Serious (-1) | Unlikely | Very Low |
| Lymph nodes dissection | 2 | 112 | 31 | 50.3 [31.16, 69.48] | Unclear | No inconsistency | No indirectness | No imprecision | Unlikely | Low |
| **Hospitalization outcomes** |  |  |  |  |  |  |  |  |  |  |
| Postoperative hospital stays | 3 | 193 | 112 | 0.47 [-0.46, 1.39] | Very Serious (-2) | Very Serious (-2) | No indirectness | Serious (-1) | Unlikely | Very Low |
| Postoperative drainage time | 3 | 193 | 112 | 0.54 [-0.33, 1.41] | Unclear | Very Serious (-2) | No indirectness | No imprecision | Unlikely | Very Low |
| **Recurrences** |  |  |  |  |  |  |  |  |  |  |
| Total recurrences | 6 | 130/656 | 42/270 | 1.27 [0.62, 2.62] | Very Serious (-2) | Very Serious (-2) | No indirectness | No imprecision | Unlikely | Very Low |
| Locoregional recurrences | 5 | 58/596 | 23/253 | 1.11 [0.46, 2.68] | Very Serious (-2) | Very Serious (-2) | No indirectness | Serious (-1) | Unlikely | Very Low |
| Distant recurrences | 5 | 77/596 | 21/253 | 1.44 [0.60, 3.48] | Unclear | Very Serious (-2) | No indirectness | No imprecision | Unlikely | Very Low |
| **Complications** |  |  |  |  |  |  |  |  |  |  |
| Total complications | 5 | 227/555 | 72/231 | 1.28 [1.04, 1.59] | Low | Serious (-1) | No indirectness | No imprecision | Unlikely | Very Low |
| Severe complications | 1 | 33/279 | 11/90 | 0.97 [0.51, 1.83] | Low | No inconsistency | No indirectness | No imprecision | Unlikely | Low |
| 90-day Mortality | 4 | 424/19402 | 19/1130 | 1.23 [0.77, 1.96] | Low | No inconsistency | No indirectness | No imprecision | Unlikely | Low |
| Conversion thoracotomy | 2 | 39/360 | 12/171 | 1.31 [0.69, 2.49] | Low | No inconsistency | No indirectness | No imprecision | Unlikely | Low |
| Pulmonary complications | 2 | 40/174 | 24/112 | 1.19 [0.75, 1.87] | Low | Serious (-1) | No indirectness | No imprecision | Unlikely | Very Low |
| Cardiac complications | 1 | 5/81 | 7/81 | 0.71 [0.24, 2.16] | Low | No inconsistency | No indirectness | No imprecision | Unlikely | Low |
| Re-operation | 2 | 1/141 | 2/98 | 0.50 [0.05, 5.41] | Low | No inconsistency | No indirectness | No imprecision | Unlikely | Low |
| Re-admission | 1 | 1/81 | 2/81 | 0.50 [0.05, 5.41] | Low | No inconsistency | No indirectness | No imprecision | Unlikely | Low |
| Atrial fibrillation | 2 | 34/153 | 9/48 | 1.25 [0.67, 2.34] | Very Serious (-2) | Very Serious (-2) | No indirectness | Serious (-1) | Unlikely | Very Low |
| Air leak >5days | 3 | 31/205 | 8/62 | 1.20 [0.58, 2.46] | Unclear | Very Serious (-2) | No indirectness | No imprecision | Unlikely | Very Low |
| Postoperative bleeding | 2 | 1/153 | 0/48 | 1.02 [0.04, 24.44] | Low | No inconsistency | No indirectness | No imprecision | Unlikely | Low |
| Acute renal failure | 1 | 1/93 | 0/31 | 1.02 [0.04, 24.44] | Very Serious (-2) | Very Serious (-2) | No indirectness | Serious (-1) | Unlikely | Very Low |
| Urinary retention | 1 | 2/93 | 0/31 | 1.70 [0.08, 34.52] | Low | No inconsistency | No indirectness | No imprecision | Unlikely | Low |
| Acute myocardial infarction | 1 | 1/93 | 0/31 | 1.02 [0.04, 24.44] | Very Serious (-2) | Very Serious (-2) | No indirectness | No imprecision | Unlikely | Very Low |
| Embolism | 1 | 0/93 | 0/31 | 0.11 [0.00, 2.72] | Very Serious (-2) | Very Serious (-2) | No indirectness | Serious (-1) | Unlikely | Very Low |
| Chylothorax | 1 | 2/52 | 0/14 | 1.42 [0.07, 27.91] | Unclear | Very Serious (-2) | No indirectness | No imprecision | Unlikely | Very Low |
| Empyema | 2 | 0/145 | 0/45 | Not estimable | Very Serious (-2) | Very Serious (-2) | No indirectness | Serious (-1) | Unlikely | Very Low |
| Wound infection | 1 | 0/93 | 0/31 | 0.11 [0.00, 2.72] | Low | No inconsistency | No indirectness | No imprecision | Unlikely | Low |

**Abbreviations:** OS: overall survival; OSR: overall survival rate; DFS: disease-free survival; DFSR: disease-free survival rate; CI: confidence interval; No.: number.

^a^ Differences: hazard ratio (HR) for OS and DFS; risk ratios (RR) for OSR, DFSR, recurrences and complications; mean difference (MD) for operative outcomes and hospitalization outcomes.

^b^ Risk of bias assessed using the Newcastle-Ottawa Scale (NOS) for non-randomized studies.

^c^ Publication bias was assessed by Egger’s and Begg’s tests.
